# The Quality of Life in Patients with Spinal Cord Injury: Assessment and Rehabilitation

**DOI:** 10.3390/jcm13061820

**Published:** 2024-03-21

**Authors:** Davide Cardile, Andrea Calderone, Rosaria De Luca, Francesco Corallo, Angelo Quartarone, Rocco Salvatore Calabrò

**Affiliations:** IRCCS Centro Neurolesi Bonino Pulejo, 98124 Messina, Italy; davide.cardile@irccsme.it (D.C.); andrea.calderone95@gmail.com (A.C.); rosaria.deluca@irccsme.it (R.D.L.); angelo.quartarone@irccsme.it (A.Q.); roccos.calabro@irccsme.it (R.S.C.)

**Keywords:** spinal cord injury, quality of life, assessment, neurorehabilitation

## Abstract

**Background and Objectives:** Spinal Cord Injury (SCI) develops when the spinal cord is damaged and leads to partial or complete loss of motor and/or sensory function, usually below the level of injury. Medical advances in the last few decades have enabled SCI patients to survive after their initial injury and extend their life expectancy. As a result, the need for outcome measures to assess health and Quality of Life (QoL) after rehabilitation is increasing. All QoL assessment measures include implicit or explicit reactions and evaluations of a person’s life characteristics. This review aims to investigate QoL and its assessment in patients with SCI and how the instruments that are used may influence rehabilitation. **Materials and Methods:** Studies were identified from an online search of PubMed, Cochrane Library, and Scopus databases. Studies published between 2013 and 2023 were selected. This review has been registered on OSF (n) 892NY. **Results:** We found that different psychological and physical aspects can positively or negatively influence the QoL of SCI patients, and the measurement of this aspect, despite the number of tools, is limited due to the lack of a universal definition of this theme and the greater prevalence of quantitative rather than qualitative tools. **Conclusions:** This review has demonstrated that clinicians and psychologists involved in SCI rehabilitation should consider tools that use high-quality standardized outcome measures to detect and compare potential differences and outcomes of interventions related to HRQoL and their relationship with the personality and functional status of the patient.

## 1. Introduction

Spinal Cord Injury (SCI) is a complex neurological condition that causes physical dependence, psychological stress, morbidity, and economic charge. It develops when the spinal cord is damaged (e.g., by trauma) and leads to partial or complete loss of motor and/or sensory function, usually below the level of injury [1]. Over the last 30 years, the global number of cases increased from 236 to 1298 cases per million population. The global incidence of SCI is estimated to have declined to 250,000–500,000 cases per year [2]. Evidence about current treatments is still scarce and, generally, treatments can only provide support for patients with lifelong disabilities [2]. Patients with SCI can experience a range of secondary physical and psychological effects, including anxiety and depression (experienced by approximately 22.2% of the population) [3] and poor QoL [4]. Immediately after the injury, stabilization of the patient becomes the priority, and therefore the patient faces many challenges on the physical, social, environmental, and psychological levels. Institutional rehabilitation provides a largely standardized supportive environment that helps SCI patients adjust to their newly acquired disabilities. Healthcare professionals work with the patient and their relatives to prepare them for their return to everyday life [4]. Several recent studies have emphasized that QoL is not strongly influenced by physical variables [5,6]. Age [7,8] and gender [9] are also weakly associated with QoL in people with SCI. Psychological assets are strong predictors of life satisfaction and welfare. They are personal characteristics and qualities that can influence how people perceive and cope with challenges. Positive emotions [7,10], high self-efficacy [10,11], optimism [12], hope [7,10], and coherence [10,13] were shown to be positively associated with improved QoL; psychological dynamics such as appraisal and the coping strategies used by SCI patients also significantly predict QoL over time [14]. Medical advances in the last few decades have enabled SCI patients to survive after their injury and extend their life expectancy [15]. As a result, the need for outcome measures to assess health and QoL after rehabilitation is increasing [16,17]. All QoL assessment measures include implicit or explicit reactions and evaluations of a person’s life characteristics (outcomes). Therefore, the distinction between whether a measure is based on an ‘objective’ or ‘subjective’ view depends on (1) whose expectations and assessments are used and (2) which reaction and/or evaluation was made explicit [18]. Some of the tools that can be used to assess QoL in patients with SCI are the following: Satisfaction With Life Survey [19]; Sense of Well-Being Index [20]; World Health Organization Quality of Life [21]; Quality of Life Index [22]; Quality of Life Profile for Adults with Physical Disabilities [23]; Short Form 36 [24]; Short Form 12 [25]; Short Form 6-Disability [26]; Short Form 36 Veterans/SCI [27]; Sickness Impact Profile [28]; Patient-Reported Impact of Spasticity Measure [29]; Quality of Well-Being Questionnaire-SA [30]. The SCI population also includes a significant number of people with pre-existing mental health disorders (MHDs). The MHDs listed in the literature include depression, personality disorders, schizophrenia, drug and alcohol abuse, and mood disorders [31]. Two plausible rationales underlie the elevated risk of SCI among individuals with a history of MHDs. A substantial proportion of SCI cases in this demographic stem from suicide attempts, with a significant subset of these instances involving individuals diagnosed with MHDs [32]. This substantiates the correlation between the presence of MHDs and suicidal behaviors. Notably, the favored modus operandi for suicide attempts is ‘jumping,’ potentially elucidating the heightened prevalence of such attempts in individuals with schizophrenia compared to the general population. This implies that MHDs might influence the choice of suicide method, thereby increasing the likelihood of physical harm if the attempt is unsuccessful [33]. Secondarily, indirect contributors to SCI encompass compromised concentration, a heightened propensity for risk-taking behavior, and substance abuse, all of which are intricately linked with MHDs. Recognizing this specific subgroup of SCI patients is paramount, necessitating a thorough examination of their long-term prognoses and rehabilitation outcomes. The Needs Assessment Checklist, serving as a clinically valid and reliable rehabilitation assessment tool, facilitates a comparative analysis of rehabilitation outcomes between individuals with MHDs and those without [34].

Another factor to take into consideration is the type of personality, such as Type D (distressed) Personality (TDP). This is delineated as the simultaneous presence of negative affect (NA) and social inhibition (SI). Negative affect encompasses the inclination towards experiencing adverse emotions, including but not limited to anger, resentment, discomfort, worry, and depressive feelings. Social inhibition, on the other hand, involves the apprehension of potential criticism or rejection from others, coupled with challenges in expressing oneself appropriately in social contexts. Additionally, social inhibition correlates with a propensity for negative emotions such as anxiety and depression. Transdiagnostic personality dysfunction (TDP) has been documented to exhibit a strong association with both anxiety and depression, as evidenced by previous studies [35,36]. SCI patients with TDP are likely to feel intense anxiety and distress and that can induce NA. According to the literature, therefore, individual differences in psychological factors can significantly influence recovery times, functional outcomes, and physical performance in SCI. Consequently, evaluating individual requirements and delivering tailored psychological support holds the potential to enhance functional autonomy and bolster the long-term Quality of Life (QoL) of individuals grappling with SCI. Given their modifiable nature, psychological factors, encompassing coping skills, health-related behaviors, and the individual’s psychological state, should be conscientiously addressed by the healthcare professional overseeing the entirety of the rehabilitation process [37,38,39]. Functional independence has been emphasized as a factor related to QoL, as it is an important indicator of independence in daily life. The practice of regular physical activity should be seen as a tool to facilitate the reintegration of people with SCI who face physical, social, and psychological challenges by developing functional independence models. In this context, positive experiences in the various domains that make up QoL enable individuals to move on with their lives [40]. Second, physical fitness is conceptualized as specific types of physical activity that are organized and structured to promote health and develop or maintain competitive skills in sports [41]. The literature on people with SCI often focuses on the potential beneficial effects of controlled physical exercise on QoL and functional independence parameters and does not address other daily activities that may contribute to an active lifestyle [42]. Exercise is an important tool when used in people with neurological paralysis. It can improve the musculoskeletal system, neural plasticity, and functional independence, promoting a good QoL for patients with SCI and a better neurorehabilitation path. This scoping review aims to investigate the QoL and its assessment in patients with SCI and how the instruments that are used may influence rehabilitation.

## 2. Materials and Methods

### 2.1. Search Strategy

**Figure 1:**
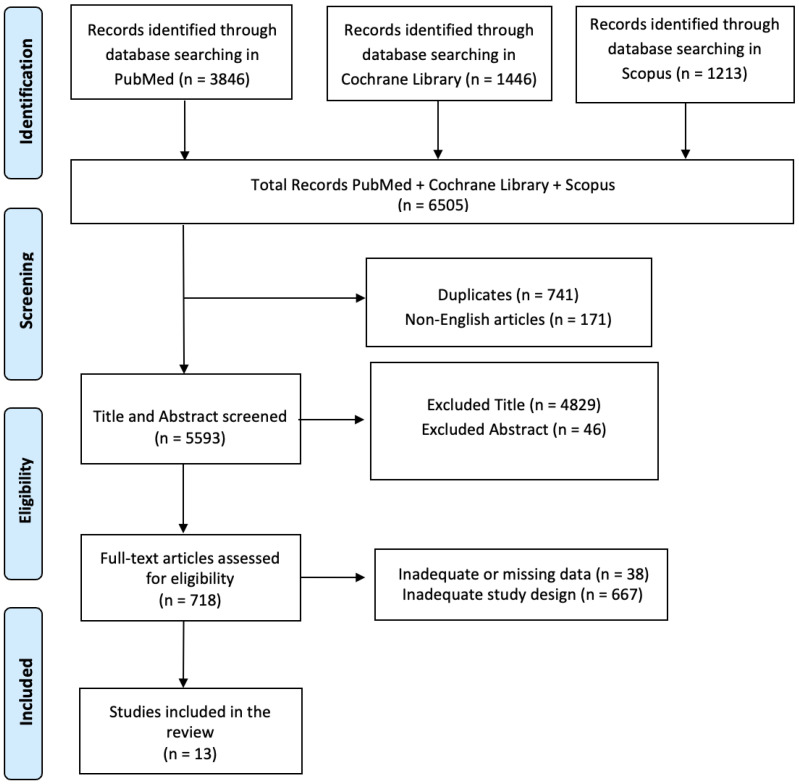
PRISMA flow diagram of the current review.

A comprehensive literature review was performed, utilizing PubMed, the Cochrane Library, and Scopus. The search strategy involved querying articles using the following string: (Title/Abstract: “Spinal Cord Injury”) AND/OR (Title/Abstract: “Personality Symptoms”) AND/OR (Title/Abstract: “Neurorehabilitation”) with a 2013/2023 search time range. The PRISMA flow diagram was implemented to delineate the sequential progression of stages (identification, screening, eligibility, and inclusion) in the compilation and evaluation of eligible studies, as depicted in Figure 1. Titles and abstracts were independently scanned and retrieved from database searches. The suitability of the article was then assessed according to the defined inclusion criteria. Ultimately, we received all titles and abstracts whose full texts met the criteria for inclusion. To mitigate the risk of bias, multiple expert teams collaborated, jointly selecting articles, independently analyzing the data, and engaging in discussions to address any disparities that arose. Disagreements between reviewers were resolved by consensus. This review was registered on OSF (n) 892NY.

### 2.2. PICO Evaluation

The search term combinations were defined using a population, intervention, comparison, outcome (PICO) model. The population was limited to patients with moderate to severe SCI; the intervention included all innovative approaches, protocols, and assessment tools used to measure and understand the psychological and contextual factors and aspects that influence QoL and rehabilitation; the comparison was evaluated considering the different tools available to measure QoL in patients with SCI, both before and during a course of psychological and motor rehabilitation; and the results included any improvements in the sensitivity of the tools used to identify the functional, motor, and psychological factors shown by patients before and after the injury.

### 2.3. Inclusion Criteria

A study was included if it described or investigated QoL and its assessment in patients with SCI and how these instruments may influence rehabilitation. The review included only articles written in English. Studies describing or investigating the functional assessment of these patients were also included. We only included studies conducted in human populations and published in English that met the following criteria: (i) original or protocol studies of any type and (ii) articles that presented some instrument assessment and functional status information that could influence the QoL and rehabilitation of patients with an SCI diagnosis.

### 2.4. Exclusion Criteria

Studies were excluded if there was a lack of data or a lack of information about or description of the QoL and its assessment in patients with SCI during the rehabilitation process. Systematic, narrative, or integrated reviews were also excluded, but reference lists were checked and added if necessary. All articles written in languages other than English were excluded.

### 2.5. Assess Quality of Included Studies—Risk of Bias

The Newcastle–Ottawa Scale (NOS) was used to assess each study (Table 1), following the criteria of the Cochrane Non-Randomized Studies Methods Working Group. The NOS was adapted to assess the methodological quality of non-randomized interventional studies. The evaluation includes key areas such as subjects’ selection, the comparability of groups, and the evaluation of outcomes. The NOS allows for a systematic assessment of potential bias, offering insights into the strengths and limitations of the reviewed studies.

## 3. Results

A total of 6505 articles were found in the selected databases. A total of 741 articles were duplicates, and so they were excluded after screening. A total of 171 articles were removed because they were not written in English, and 4875 articles were excluded based on the title and abstract screening. Finally, 705 articles were removed based on the screening for inadequate study designs and untraceable articles (Figure 1). Thirteen research articles met the inclusion criteria, and were therefore included in this review. A survey of these studies is shown in Table 2.

The articles described in this review investigated the QoL and its assessment in patients with SCI and how these instruments may influence rehabilitation. QoL and a well-being assessment of patients with SCI were analyzed in six articles [43,44,45,46,48,49]. The methods used to perform clinical evaluation of QoL and symptoms of SCI have been described in three articles [47,51,52]. The relationship between functional status, rehabilitation, and comorbidities was explained in four articles [50,53,54,55].

### 3.1. QoL and Assessment in Patients with SCI

The QoL and the tools available for evaluation are essential to establish an adequate rehabilitation path for patients with SCI. A summary of these tools can be found in Table 3. In one study, it was found that QoL in SCI patients can be influenced by factors such as spirituality and depression; in a sample of 210 people, approximately 26% had major depressive disorder. Functional Assessment of Chronic Illness Therapies—Spiritual, as measured by spirituality, has proven to be an important determinant of QoL [43]. A second study demonstrated that WHOQOL-BREF and PWI prescribed injury-level group classification, while WHOQOL-BREF prescribed environment group classification. Neither questionnaire differentiated between injury type. In the early post-acute phase of rehabilitation, there was no significant difference in QoL scores between tetraplegia and paraplegia, while QoL scores were significantly higher for paraplegia in the long-term environment group. In the early post-acute phase of rehabilitation, QoL was significantly higher for paraplegia than for tetraplegia [44]. A multicentered study proved that DMSE constitutes a psychological resource correlating with higher levels of participation and life satisfaction after SCI. The UW-SES-6 is a brief and simple measure of this psychological resource [45]. A prospective observational registry cohort study revealed the complex interactions and lasting effects of health conditions that negatively impact functioning, Health-Related Quality of Life (HRQoL), and life satisfaction following SCI. A worse health status was negatively correlated with worse mental health and positively correlated with lower functioning. Being married and having higher functioning had a positive effect on the Lisat-11 test, while a worse health status had a negative effect [46]. A longitudinal study proved that people with chronic SCI may be vulnerable to mental health problems even if they previously demonstrated good resilience. Furthermore, subjective well-being after SCI may not be as stable as the general QoL literature examining genetic and personality associations with subjective well-being. No statistically significant distinctions in age or duration since injury were observed between individuals reporting noteworthy emotional symptoms and those who did not. Furthermore, there was an absence of any discernible systematic alterations in health status. [48]. A cross-sectional study showed that, in a sample of 105 patients, 39% of people with SCI had TDP and the mental component of HRQoL was associated with TDP in people with SCI. Vitality, emotional role, and mental health scores were also significantly lower; TDP primarily predicted the mental health component of the SF-36; NA was a significant predictor of mental health, especially vitality and mental health; and the mental health component of the SF-36 was a significant predictor of the mental health component of HRQoL [49]. In another article, a longitudinal measurement of invariance was conducted, showing that, in terms of measurement and validation tools, the SCI-QoL-BDS represent a valid measure for assessing the QoL of people undergoing inpatient rehabilitation for their first SCI/disability. The SCI-QoL-BDS consists of three items assessing general life, physical, and mental state. The intercepts of all items, except satisfaction with physical health, are invariant over time, indicating the partial invariance of the SCI-QoL-BDS intercepts [47]. Another study showed that a multimodal pain assessment approach combining clinical examination, quantitative sensory testing, blood biomarkers, and an assessment of psychosocial factors at various time points is effective and functional in the early stages of rehabilitation after SCI. More specifically, the SCIPI questionnaire is effective in differentiating nociceptive pain from neuropathic pain, which progressively increases in severity over time [51]. One last article demonstrated that, by adding three additional items measuring arm and shoulder function to the NRS, expanding the scoring criteria to full recovery, and using the new NRS score, estimates of neuromuscular functional recovery after SCI were improved across a wide range of levels and severities.

The NRS score improves neuromuscular recovery estimates across a wide range of SCI levels and severities and furnishes a heightened level of sensitivity and a comprehensive metric for clinical practice and research pertaining to functional recovery following SCI. The NRS score is a more comprehensive measure than other measures commonly used as endpoints for SCI rehabilitation [52].

### 3.2. Rehabilitation, Comorbidities, and Functional Status in SCI

The assessment of comorbidities and the functional status of the patient with SCI are two aspects that should be paid attention to during the diagnosis and before setting up a rehabilitation program. One study found that the commonly used comorbidity indicators do not reflect the extent of comorbidity in the SCI rehabilitation population. Furthermore, ICD-10 CM does not accurately capture the comorbidities that are prevalent in this population and SCI sequelae are coded with high frequency. Functional status is a better predictor of readmission than comorbidities in many inpatient rehabilitation populations, including SCI ICD coding data, but provides less information on disease severity and clinical instability. The existence of conditions influencing functional status could serve as a more reliable indicator of clinical status. Consequently, it may exhibit enhanced sensitivity in capturing patient characteristics that exert an impact on the outcomes. [50]. From a functional perspective, SEM was used to examine the possible influence of mental function on the relationship between physical structure, physical function, and activity, and structural models of depression, optimism, and self-esteem showed that pain had a significant indirect effect on independence in ADL activities. In the structural model of anxiety, group differences were found in the etiologic group. Since pain was the only physical function that had an indirect effect on independence in ADL activities in the structural model for depression, optimism, and self-esteem, it is worth re-examining the relationship between pain and these mental functions in more detail, in combination with other pain items, such as clinical pain records [53]. Another study found that, in patients with SCI undergoing initial rehabilitation, functional trajectories were categorized into four different classes. Given a sample of 748 individuals, the mean functional trajectories estimated by class were defined as stable high functioning (*n* = 307; 41.04%), early functional recovery (*n* = 39; 5.21%), moderate functional recovery (*n* = 287; 38.37%), and slow functional recovery (*n* = 115; 15.37%), in order of the identified classes. Trajectory studies of outcomes such as life satisfaction and employment status showed that independence in ADL performance, as assessed by the FIM, was a trajectory predictor for each class [54]. One last article shows that complications are more common in patients with SCI. The presence of complications negatively affects functional status at discharge and length of hospital stay, increasing the risk of institutionalization. Patients without complications had significantly better functional status at admission and discharge compared to patients with complications [55].

## 4. Discussion

Our review aimed to analyze the QoL and its assessment in patients with SCI and how these instruments may influence rehabilitation. The studies included in this review have demonstrated that the QoL of SCI patients can be influenced by factors such as spirituality and depression, and Functional Assessment of Chronic Illness Therapies measuring spirituality were proven to be an important determinant of QoL. Furthermore, in the early post-acute phase of rehabilitation, QoL is significantly higher in paraplegia than in tetraplegia [43,44]. It has also been shown that DMSE is a psychological resource associated with greater engagement and life satisfaction after SCI, while a worse health status was negatively correlated with worse mental health and positively correlated with lower functioning. Moreover, being married and having good functioning has a positive effect on the Lisat-11 test, while a worse health status has a negative effect [45,46]. Some articles also suggest that chronic SCI patients may be more prone to mental health problems even if they were previously more resilient; the mental component of HRQoL in SCI patients was associated with TDP. In addition, TDP primarily predicted the mental health component of the SF-36, while NA is an important predictor of mental health, especially vitality and mental health [48,49]. In terms of the methodological and clinical tools used to assess QoL in these patients, the SCI-QoL-BDS was found to be a consistent and valid measure to assess this aspect in patients entering hospital rehabilitation for the first time after SCI/disability. Furthermore, a multifaceted pain assessment approach combining clinical examination, quantitative sensory testing, blood biomarkers, and an assessment of psychosocial factors at various time points is effective and functional at the early stages of rehabilitation after SCI, and the NRS score appears to improve pain prediction. Neuromuscular recovery is a more sensitive and comprehensive indicator in clinical practice and research on functional recovery after SCI due to the wide range of levels and severity [47,51,52]. Articles on rehabilitation, comorbidities, and functional capacity in these patients show that ‘ICD-10 CM fails to accurately capture comorbidities that are common in this population. Functional status is a better predictor of readmission than comorbidities in many inpatient rehabilitation populations. From a functional perspective, pain has been proven to have a significant indirect effect on independence in ADL activities. Furthermore, trajectory studies on outcomes such as life satisfaction and employment status have shown that independence in ADL performance, as assessed by the FIM, is a predictor of the trajectory of development. In addition, the presence of comorbidities harms functional status at discharge, in addition to increasing the length of stay, and increases the risk of institutionalization [50,53,54,55]. The literature shows that the majority of studies examining QoL after SCI have predominantly embraced a quantitative methodology. Researchers have employed various tools, including single-item rating scales, multi-item rating scales (addressing overall life satisfaction), and multi-item questionnaires (with items gauging satisfaction with specific aspects of life) [56]. However, the inconsistency in approaches poses challenges for obtaining a comparison and agreement across studies.

Beyond the methodological disparities, quantitative investigations into QoL encounter inherent challenges. Firstly, attempts to quantify qualitative experiences tend to blur the distinctions between quantitative and qualitative realms [57]. Additionally, the delineation of categories deemed relevant in conventional research is influenced by the choices of “experts” [58,59], inherently reflecting their values and cultural context [60,61]. Such choices are not truly “neutral” [62] or “objective” [60].

Fundamentally, the complexity of assessing QoL arises from the challenge of comprehending a life distinct from one’s own and determining the value attributed to that life. The scientific literature indicates that individuals with high tetraplegia exhibit suboptimal performance on scales employing objective factors to measure QoL. Instead, the subjective experience of life emerges as more pivotal than external expressions.

Understanding this subjective experience requires research methods that can explore both the content and context of life [63]. The scientific literature presents a significant association between QoL and gender, education level, injury classification, level of injury, and the presence of pain or pressure on the injury. People with incomplete injuries and paraplegia have been reported to have better self-care skills compared to those with complete injuries, tetraplegia, and quadriplegia. This may lead to an improved QoL as they are not completely dependent on caregivers. Similarly, higher levels of injury and lower QoL are observed in people with complete injuries. Furthermore, people with pressure ulcers have a lower QoL due to prolonged bed rest, leading to physical, social, psychological, and environmental life limitations [64]. From the perspective of personality and possible protective factors against a worse QoL, physical and positive behavior exercises are observed to have an effect. Several psychological tests have shown that wheelchair athletes have higher levels of self-satisfaction, a stronger self-image, less suicidality, and greater independence than relatively less physically active people with an SCI [65,66,67]. Research proved that the majority of SCIs are secondary to trauma and approximately 85% of cases occur in men. Therefore, men who develop an SCI as a result of trauma may share some specific personality traits. In general, the data suggest that men with an SCI tend to be pragmatic and physically oriented, have difficulty communicating their thoughts and feelings, solve abstract problems, and dislike activities that require intense interpersonal interaction. In middle age, they tend to continue to seek stimulation, remain interested in physically challenging and adventurous activities, and become less intellectually curious, persistent, and achievement-oriented [68]. They prefer to work outdoors, with objects (tools and machines), rather than with ideas and people. Other data suggest that people in this group are visual and kinesthetic learners and prefer to learn by exploring in an actively challenging environment [69]. They tend to avoid physical closeness and can be distant from other individuals. Their personality traits do not simply reflect their youthful adventurousness but rather are enduring characteristics. A tendency to use an evasive and impulsive approach to problem-solving is observed more often [70]. Some of the differences observed between men with SCI and the normative population may reflect their pre-injury personality. Some SCI patients are ambivalent about counseling or meeting with mental health providers. Young men in particular may need introspection and may not fit with traditional psychological approaches that emphasize verbal interaction [71]. Regarding the rehabilitation aspect, there are many psychological interventions, including psychotherapy, that can be used to support optimal functioning and QoL in SCI patients. For example, cognitive behavioral therapy includes interventions to improve beliefs, attitudes, and thought patterns that support positive emotions and are compatible with adaptive functioning. Cognitive restructuring helps patients to identify overly negative and distorted thought patterns and formulate and focus on more realistic and productive thought processes. Cognitive restructuring strategies are effective in reducing the cognitive distortions of catastrophizing views and associated anxiety and increasing self-efficacy [72]. There are also motivational interviewing techniques that increase people’s intrinsic motivation to make positive changes by helping patients identify desired outcomes, highlighting discrepancies between current behavior and the behavior needed to achieve the desired outcomes, and building their confidence so that they believe change is possible. Specific strategies include using active listening to build a rapport, facilitating the clarification of goals, guiding the person to expand the range of perceived options for achieving those goals, eliciting commitment to change, and affirming positive movement in that direction [73,74,75].

This scoping review had several strengths. It is based on evidence from longitudinal observational populations and cross-sectional studies with large sample sizes. It includes an analysis of the instrument used for the assessment of QoL in SCI patients. We also identified data gaps in many areas, hopefully providing information for future research. The main limitation of the present study is the few papers that meet the inclusion criteria, as we included only thirteen articles that explored QoL and its evaluation in patients with SCI and only four of them focused on the relationship between functional status, rehabilitation, and comorbidities. This, besides the heterogenous methodology and samples, prevents us from gathering robust evidence on this important topic. Three databases were also used, and the articles were restricted by date, so it is possible that important evidence was omitted. It is necessary to conduct studies on these patients to evaluate the QoL of the family and the patients regarding their experience with the disease and impairment. There is also a need for the development of a standardized qualitative tool that assesses QoL in patients with SCI to better understand their psychological needs. There are several promising tools for measuring QoL. Unfortunately, due to the lack of consistent findings and definitions, knowledge about QoL in people with SCI is still limited. In this review, we attempt to make comparisons between different QoL measures, and it should be noted that we are trying to compare different QoL measures based on different definitions. Further research is needed on the universal definition of this topic and the relevance and impact of different aspects of the lives of people with SCI regarding QoL.

In conclusion, this review shows that various psychological and physical elements can positively or negatively affect the QoL of people with SCI, that there is no universal definition of this issue, despite several available assessment tools, and that the prevalence of quantitative tools over qualitative ones limits the measurement of this element. The methodological aspects of QoL research on patients with SCI need to be improved. Many scientists and clinicians developed their scales during their research. It is important to use scales with proven reliability and validity. If a new scale is developed for a specific study, its psychometric properties need to be determined and tested. It is important to use high-quality, standardized outcome measures to detect and compare the results of interventions. Given the few studies included in our work, the conclusions that can be drawn are preliminary and the current evidence requires further investigation. Clinicians and psychologists taking part in SCI rehabilitation should consider potential differences regarding dissimilar personality traits and HRQoL. Further studies that develop and apply psychological interventions and follow person-specific goals could be of use, as well as more studies on the pre-morbid personality of these patients.

## Figures and Tables

**Table 1:** Newcastle–Ottawa Scale results for each study involved in this review.

Study	Selection	Comparability	Outcome Assessment	Total Score
Wilson et al., 2017 [43]	2	2	1	5
Zwecker et al., 2022 [44]	2	1	2	5
Cijsouw et al., 2017 [45]	2	1	1	4
Rivers et al., 2018 [46]	2	2	2	6
Kunz et al., 2022 [47]	2	1	1	4
Migliorini et al., 2013 [48]	0	1	2	3
Eroglu et al., 2022 [49]	1	1	2	4
Huang et al., 2020 [50]	3	2	1	6
Capossela et al., 2023 [51]	0	1	2	3
Harkema et al., 2016 [52]	1	2	2	4
Hodel et al., 2020 [53]	2	2	1	5
Hodel et al., 2021 [54]	3	1	1	4
Scivoletto et al., 2020 [55]	1	1	1	3

**Table 2:** Summary of studies included in the research.

Author	Aim	Study Design/Intervention	Treatment Period	Sample Size	Outcomes Measures	Main Findings	Study Limitations	Statistical Analyses
Wilson et al., 2017 [43]	To determine the relationship between mental well-being, demographic characteristics, QoL, and depressive symptoms after SCI.	Randomized controlled trial.	Not specified.	210 individuals with SCI.	PHQ-9, FACIt-Sp, Neuro-QOL, PAWB, PANAS.	Spirituality, as measured by FACIT-Sp, is strongly associated with QoL and the likelihood of MDD. Spirituality assessments should be carried out using more traditional psychological measures for better treatment.	This study is limited by its sample. It is unclear whether the dynamics of QOL and depression in this sample reflect the dynamics of the SCI population as a whole. In addition, this study does not address the causal nature of the observed relationships.	Descriptive statistics and tests of association (Pearson correlation, *t*-test, analysis of variance, chi-square test, etc.) were used to examine the relationship between sample attributes and survey results, and ultimately assess which attributes should be included in the multivariate analysis as adjustment factors.
Zwecker et al., 2022 [44]	To evaluate the unmediated relationship between neurologic impairment and QoL in patients with SCI in early post-acute and long-term rehabilitation settings.	An observational, prospective, cross-sectional study.	Not specified.	156 adults with SCI.	WHOQOL-BREF, ASIA, AIS, SWLS, LISAT-9, PWI.	WHOQOL-BREF differentiated between tetraplegic and paraplegic groups, but not between complete and incomplete injury groups; QoL in the early post-acute rehabilitation period was significantly higher in paraplegics than in tetraplegics.	The generalizability of the results of this study is limited by the sampling technique. This is because only SCI participants who voluntarily attended outpatient follow-up were included in the chronic group. These patients are more likely to have medical comorbidities that may unidirectionally affect HRQoL.	Binomial Logistic Regression Analysis was performed to determine which questionnaire score most effectively differentiates between groups based on injury level (paraplegic vs. quadriplegic), setting (inpatient vs. outpatient), and injury type (complete vs. incomplete). The dependent variable in this analysis was the scale used for classification, and the independent variable was the QoL questionnaire.
Cijsouw et al., 2017 [45]	To examine DMSE and its correlates across different life domains in a large sample of Dutch people with long-term SCI.	Cross-sectional study.	Between November 2011 and February 2014.	261 individuals.	UW-SES-6.	DMSE is a psychological resource associated with higher engagement and life satisfaction after SCI. The UWSES-6 is a simple and easy-to-use measure of this psychological resource.	The limitations of this study stem from the inclusion criteria. The study sample predominantly consists of patients with traumatic complete SCI, and these patients acquired an SCI at a relatively young age. This affects the extent to which the results of this study can be generalized to all patients with SCI.	The relationships between UW-SES-6 scores and other variables were analyzed using t-tests or analysis of variance for categorical variables and Pearson correlation coefficient for continuous variables. A correlation coefficient of 0.30 was interpreted as weak, 0.30–0.50 as moderate, and up to 0.50 as strong.
Rivers et al., 2018 [46]	To analyze the relationship between injury, demographic and environmental factors, and function, HRQoL and life satisfaction in persons with traumatic SCI.	Prospective observational registry cohort study.	From 2004 to 2014.	340 participants.	FIM, HRQoL, PCS, MCS, LISAT-11.	Higher age, higher severity of injury, cervical spine injury, and worse health status had a negative effect on FIM motor scores, while employment had a positive effect. Higher age, lower level of education, more serious injury and worse health status were negatively correlated with PCS. More health conditions were negatively correlated with lower MCS but positively correlated with poorer functioning. Being married and having higher functioning had a positive impact on Lisat-11, while worse health status had a negative impact.	Model results only apply to the 340 of the 580 individuals who met the study inclusion criteria and for whom all outcome data were available; outcome data were not imputed because there was a sufficient sample size to use only real-world data.	Path analysis was performed using Mplus version. Starting from a saturated model, a backward selection process was used to remove associations of non-significant variables. The goodness of fit of the model was assessed with five fit indices: the chi-square test (*p* > 0.05 is considered a good fit), root mean square error of approximation (<0.05 was considered a close fit, and an upper value of 0.080 was considered a reasonable fit), the 39 comparative fit index and the Tucker–Lewis Index (value > 0.95 × 1.0 indicates a good fit of 40, where the model is close to the study data), and standardized root mean square residual difference (<0.08 recommended).
Kunz et al., 2022 [47]	Validation of the internal consistency and longitudinal measurement invariance of the SCI-QoL-BDS in SCI and disability patients undergoing initial inpatient rehabilitation.	Prospective observational cohort Study.	Between May 2013 and January 2021.	218 participants.	SCI-QoL-BDS.	The SCI-QoL-BDS is a consistent and valid measure that is used to assess QoL in people undergoing inpatient rehabilitation for the first time due to SCI/disability. However, to account for potential variations in response, it is recommended to use a latent variable framework rather than a mean score when examining longitudinal changes in the measures.	The longitudinal measurement invariance of the SCI-QoL-BDS has been examined in the inpatient rehabilitation of SCI patients. Therefore, it is unclear whether similar results would have emerged if measurement time points in community settings had been included. The post-hoc study of partial invariance at the intercept level is a data-driven approach. When comparing participant and non-participant characteristics with the existing data, the current study showed a slight selection bias.	Frequency statistics were used to describe the characteristics of the study sample and the SCI-QoL-BDS items and total scores. To test the internal consistency of the SCI-QoL-BDS, McDonald’s omega, Cronbach’s alpha, and corrected item–total correlations were calculated for each time point. Item–total correlations were calculated for each time point (T1, T2, T3). The longitudinal measurement invariance of the SCI-QoL-BDS was examined using cross-factor analysis.
Migliorini et al., 2013 [48]	To test whether people whose subjective well-being returns to a normal homeostatic range after SCI are more resilient and therefore less at risk of emotional distress in the long term.	Longitudinal study.	Not specified.	Not specified.	COMQoL-A5), PWI, DASS-21.	Patients with chronic SCI may be vulnerable to mental health problems even if they have previously demonstrated good resilience and subjective well-being. Subjective well-being may not be stable after SCI.	The small sample size is a serious limitation of the study and restricts the ability to draw firm conclusions from the study results. In addition, the sampling method used for the purpose of this study may also have influenced the study results.	Cronbach’s alpha coefficient is 0.84. Subjective well-being was calculated as the average of the seven subjective QoL domains and converted into a percentage of the highest score of the scale.
Eroglu et al., 2022 [49]	The aim of this study was to examine the relationship between TDP and functional outcomes, HRQoL and neuropathic pain in people with SCI using binary and continuous analysis methods.	Cross-sectional study.	12 months.	105 persons with SCI.	FIM, HRQoL, DS-14, LANSS.	The mental component of HRQoL is associated with Type D in SCI patients in both analyses. Identifying potential differences is useful for the development and implementation of individual-specific goals in SCI rehabilitation.	The cross-sectional design of the study does not provide information on the course of type D patients over time. The small number of participants treated at a single center may limit the generalizability of the results to the entire population of SCI patients. The collection of limited demographic variables is also a limitation.	Categorical variables are expressed as the number and percentage of cases. Continuous data for normal distributions are presented as mean (standard deviation) unless otherwise stated. Chi-square tests were used to compare data on gender, injury severity, and the level and presence of neuropathic pain between D and non-D groups.
Huang et al., 2020 [50]	Examine whether commonly used comorbidity indicators and CMS comorbidities capture the comorbidities of acute trauma and non-traumatic SCI inpatient rehabilitation patients.	Retrospective cross-sectional study.	From 10 October 2015 to 31 December 2017.	833 inpatients.	ICD-10-CM.	Commonly used comorbidity indices do not reflect the extent of comorbidity in SCI rehabilitation populations. This study suggests that alternative indicators are needed to capture the complexity of this population.	UDSMR records represent discharges from rehabilitation, not individual persons. This implies that an individual who was discharged more than once from an IRF would be represented more than once.	Descriptive statistics of demographic and medical data were calculated using Stata version 15.1 (StataCorp LLC, College Station, TX, USA). The study population was divided into traumatic and non-traumatic etiologies. For the total population, traumatic, and non-traumatic groups, comorbidity frequencies for each ICD-10-CM code were calculated as a percentage of the total number of discharges in each group. Comorbidity frequencies of above 1% were reported.
Capossela et al., 2023 [51]	This study will evaluate the feasibility of a multimodal pain assessment protocol in rehabilitation after SCI.	Cohort study.	From September 2017 to December 2018.	53 patients	CW/QST, SCIPI.	The SCIPI has been shown to be effective in differentiating between nociceptive and neuropathic pain, with a progressive increase in severity over time. Descriptive statistical analysis showed no difference in QoL, but stress and depression decreased and anxiety increased after initial rehabilitation.	Successful SCI-related pain research requires the coordination of recruitment settings, time resources, and assessment protocols. Questionnaires were not always completed by patients.	In gene expression and immunoassay analyses, the nonparametric Wilcoxon signed-rank test was used as the variable of interest to compare T1 and T4.
Harkema et al., 2016 [52]	Explore the features of the expanded NRS, introduce and evaluate new scoring methods, and examine its relationship to other SCI outcome measures.	Prospective observational study.	5 training sessions per week.	152 participants.	ISNCSCI, NRS, 6MWT, 10MWT, MFR, BBS.	The new NRS score responded most to changes related to motor training. The expanded NRS appears to be a valuable tool for measuring functional recovery from SCI.	The analysis does not represent a formal psychometric evaluation of the properties of the updated NRS. In particular, the added arm and shoulder items require reliability testing and a formal NRS validity assessment rather than principal component analysis.	Baseline demographic and clinical characteristics were summarized as numbers and percentages for categorical data, means and standard deviations for continuous data. Associations between NRS scores, NRS stages, NRS empirical subscale scores, and other continuous functional and clinical measures were assessed by calculating marginal Pearson correlation coefficients for clustered data.
Hodel et al., 2020 [53]	To examine the relationship between activity, physical structure, and function, as well as their association with etiology, age, and gender during initial rehabilitation discharge in patients with SCI.	Cross-sectional study.	Not specified.	390 participants with SCI.	ICF, ADL.	The structural model for optimism showed a good fit across all indicators, whereas the models for anxiety, depression, and self-esteem showed conflicting fit indicators for each.	Invariance in the measurement model and group differences in the structural model could not be detected. Small sample size.	SEM was used to examine the indirect effects of physical structure and function on independence in ADL performance through the mental functions of anxiety, depression, optimism, and self-esteem, separately for each mental function.
Hodel et al., 2021 [54]	To identify functional trajectory classes in SCI patients undergoing initial rehabilitation after injury and to examine the potential determinants of class membership to inform clinical planning of the rehabilitation process.	Longitudinal cohort study.	Between May 2013 and September 2019.	748 individuals.	SCIM III.	The mean predicted functional trajectories by class were stable high functioning (*n* = 307; 41.04%), early functional recovery (*n* = 39; 5.21%), moderate functional recovery (*n* = 287; 38.37%), and slow functional recovery (*n* = 115; 15.37%). Multinomial logistic regression results showed that age, level of injury, severity of injury, and ventilator assistance were strong predictors that differentiated the functional trajectory classes defined in this sample.	Selection bias may have occurred. This was due to the exclusion of individuals with fewer than two observations. Country-specific differences in clinical rehabilitation practices (e.g., availability, appropriateness, comprehensiveness, and duration of inpatient rehabilitation) may further limit the generalizability of the results.	LPMMs were used to determine the number of different functional trajectory classes within the current sample of individual interval-based SCIM III total score trajectories.
Scivoletto et al., 2020 [55]	The aim of the study was to evaluate the impact of complications at admission on the functional status of patients with SCI.	Retrospective cohort study.	Between 1996 and 2020.	207 patients.	SCIM, RMI, WISCI.	Patients with complications on admission are more likely to have traumatic lesions. Patients without complications had significantly better functional status at admission and discharge compared to patients with complications.	The study was initiated in 1996 and only the associated lesions and complications were included. Both associated lesions and complications were simply classified as present/absent, without any assessment of severity that could make a clear difference.	Descriptive data analysis: descriptive values expressed as mean + SD were provided for all continuous clinical data. Paired data were analyzed by paired *t*-test; McNemar’s chi-square test was applied to assess split differences.

Legend: Quality of Life (QoL); Spinal Cord Injury (SCI); Functional Assessment of Chronic Illness Therapies—Spiritual (FACIT-Sp); Major Depressive Disorder (MDD); Patient Health Questionnaire-9 (PHQ-9); Quality of Life in Neurological Disorders (Neuro-QOL); Positive Affect and Well-Being Short Form (PAWB); The Positive and Negative Affect Schedule (PANAS); World Health Organization Quality of Life Assessment-BREF (WHOQOL-BREF); American Spinal Injury Association (ASIA); Impairment Scale (AIS); Satisfaction with Life Scale (SWLS); Life Satisfaction Questionnaire (LISAT-9); Personal Well-Being Index (PWI); Disability-Management Self-Efficacy (DMSE); Self-Efficacy Scale-Short Form (UW-SES-6); Health-Related Quality of Life (HRQoL); Functional Independence Measure (FIM); Physical Component Score (PCS); Mental Component Score (MCS); Life-Satisfaction-11 (LISAT-11); Brief Quality of Life Questionnaire—Spinal Cord Injury Quality of Life Basic Data Set (SCI-QoL-BDS); Comprehensive Quality of Life Scale—Adult v5 (COMQoL-A5); Personal Well-being Index (PWI); Depression; Anxiety & Stress Scale—short form (DASS-21); Type D personality (TDP); Type D Scale-14 (DS-14); Leeds Assessment of Neuropathic Symptoms and Signs (LANSS); Centers for Medicare and Medicaid Services (CMS); International Classification of Diseases; 10th 13 Revision; (ICD-10-CM); Inpatient Rehabilitation Facility (IRF); SCI Pain Instrument (SCIPI); Pain Clinical Workup/Quantitative Sensory Testing (CW/QST); Neuromuscular Recovery Scale (NRS); International Standards for the Neurological Classification of Spinal Cord Injury (ISNCSCI); Six Minute Walk Test (6MWT); 10 Meter Walk Test (10MWT); Modified Functional Reach (MFR); Berg Balance Scale (BBS); Structural Equation Modelling (SEM); Activity of Daily Living (ADL); International Classification of Functioning (ICF); Spinal Cord Independence Measure version III (SCIM III); Latent Process Mixed Models (LPMMs); Spinal Cord Independence Measure (SCIM); Rivermead Mobility Index (RMI); Walking Index for Spinal Cord Injury (WISCI).

**Table 3:** The subjective measures used to assess QoL following SCI.

QoL Measures in SCI Patients	Description/Structure
Subjective Tools	
Satisfaction With Life Scale (SWLS) [19]	Assess life satisfaction comprehensively, encapsulating a global perspective of an individual’s values. Due to the open-ended nature of the questions, the scale proves suitable for adults from diverse backgrounds, acknowledging the potential for varied interpretations. It is best suited for use in non-clinical populations. Subjective well-being is conceptualized as consisting of two main components: an affective/emotional component and a judgmental/cognitive component; the SWLS is designed to measure the judgmental component. It is structured into five items: a 7-point Likert Scale from “1” (strongly disagree) to “7” (strongly agree).
Sense of Well-Being Index (SWBI) [20]	Addresses a subjective index of QoL for people with disabilities during work rehabilitation, addresses successful rehabilitation beyond objective employment outcomes. It assesses QoL in people with SCI using four factors (financial, family and social, psychological, and physical well-being). It is structured into 26 items: a 4-point Likert scale from “1” (strongly disagree) to “4” (strongly agree).
World Health Organization Quality of Life (WHOQOL-BREF) [21]	An instrument that aligns conceptually with the World Health Organization (WHO) definition of Quality of Life (QoL), encompassing domains such as physical health/capacity, psychological health/well-being, social relationships, environment, overall QoL, and general health. It assesses QoL in the context of personal culture, values, personal goals, standards, and concerns. It is structured into 26 items: a 5-point Likert scale from “1” to “5”.
Quality of Life Index (QLI) [22]	The QLI is a self-report scale meticulously crafted to assess subjective QoL by gauging satisfaction across various life domains. This instrument takes into account both satisfaction and perceived importance within four distinct domains: health and functioning, psychological and spiritual well-being, social and economic aspects, and family dynamics. It is structured into 32–37 items: a 6-point Likert scale for importance and for satisfaction, from “1” (very dissatisfied) to “6” (very satisfied).
Quality of Life Profile for Adults with Physical Disabilities (QOLP-PD) [23]	A holistic approach to QoL empowers individuals, recognizing that certain QoL elements are shared by both individuals with and without disabilities, yet acknowledging that each may address these issues in unique ways. It is structured into 102 items: a 5-point Likert scale from “1” (not all satisfied) to “5” (extremely satisfied) and from “1” (not important) to “5” (very important).
Short Form 36 (SF-36) [24]	A self-administered questionnaire, filled in by the patient, which aims to quantify the state of health and measure QoL related to health. It encompasses fundamental human values pertinent to QoL and well-being by considering individual domains and incorporating two overarching global components. It is structured into 36 items and divided into two summary scores: physical and mental component.
Short Form 12 (SF-12) [25]	The SF-12 stands as a self-report outcome measure designed to evaluate the influence of health on an individual’s daily life, commonly employed as an indicator of QoL. Deriving from its predecessor, the SF-36, which originated from the Medical Outcomes Study, the SF-12 was specifically developed to alleviate response burden by offering a more concise version. It is structured into 12 items.
Short Form 6-Disability (SF-6D) [26]	A six-dimensional health state classification derived from the SF-36 includes domains such as physical functioning, role limitation, social functioning, pain, mental health, and vitality. It is structured into 11 items with a range from “the worst health state” to “perfect or full health”.
Short Form 36 Veterans/SCI (SF-36V) [27]	Version of SF-36 designed for use in the disabled population. It is structured into 36 items, with physical and mental summary scores.
Sickness Impact Profile (SIP68) [28]	A generic health status measure that incorporates specific assessments of health-related changes in behavior associated with the execution of daily activities. It is structured into 68 items that assess somatic autonomy, mobility control, mobility range, social behavior, emotional stability, psychological autonomy and communication.
Patient-Reported Impact of Spasticity Measure (PRISM) [29]	A health-related subjective well-being scale featuring seven subscales aiming to evaluate the impact of spasticity linked to Spinal Cord Injury (SCI) on Quality of Life (QoL) from the patient’s perspective. These subscales encompass ‘social avoidance/anxiety’, ‘psychological agitation’, ‘daily activities’, ‘need for help/positioning’ and ‘need for intervention’, ‘social embarrassment’, and seven additional dimensions. This scale takes into account both negative and positive aspects related to spasticity. It is structured into 41 items: a 5-point Likert Scale from “0” (never true for me) to “4” (very often true for me).
Quality of Well-Being Questionnaire-SA (QWB-SA) [30]	A questionnaire to measure HRQOL with the following specifications: symptoms and problems; mobility; physical activity; social activity. It includes an assessment of symptoms in addition to various areas of functioning. It detects changes in samples of migraineurs, cataract surgery patients, mental health populations, and arthritis patients. It is structured into 71 items.

Legend: Quality of Life (QoL); Spinal Cord Injury (SCI); Satisfaction With Life Survey (SWLS); Sense of Well-Being Index (SWBI); World Health Organization on Quality of Life (WHOQOL-BREF); World Health Organization (WHO); Quality of Life Index (QLI); Quality of Life Profile for Adults with Physical Disabilities (QOLP-PD); Short Form 36 (SF-36); Short Form 12 (SF-12); Short Form 6-Disability (SF-6D); Short Form 36 Veterans/SCI (SF-36V); Sickness Impact Profile (SIP68); Patient-Reported Impact of Spasticity Measure (PRISM); Quality of Well-Being Questionnaire-SA (QWB-SA); Health-Related Quality of Life (HRQOL).

## Data Availability

The data that support the findings of this study are not openly available due to reasons of sensitivity and are available from the corresponding author upon reasonable request.
